# Effect of Different Preconditioning Regimens on the Expression Profile of Murine Adipose-Derived Stromal/Stem Cells

**DOI:** 10.3390/ijms19061719

**Published:** 2018-06-10

**Authors:** Patrick C. Baer, Jürgen M. Overath, Anja Urbschat, Ralf Schubert, Benjamin Koch, Asanke A. Bohn, Helmut Geiger

**Affiliations:** 1 Division of Nephrology, Department of Internal Medicine III, Goethe-University, 60596 Frankfurt/M., Germany; juergen.overath@kgu.de (J.M.O.); b.koch@med.uni-frankfurt.de (B.K.); s9672561@stud.uni-frankfurt.de (A.A.B.); h.geiger@em.uni-frankfurt.de (H.G.); 2 Department of Biomedicine, Aarhus University, 8000 Aarhus, Denmark; anja.urbschat@biomed.au.dk; 3 Division of Allergology, Pneumology and Cystic Fibrosis, Department for Children and Adolescents, University Hospital, Goethe-University, 60596 Frankfurt/M., Germany; ralf.schubert@kgu.de

**Keywords:** stem cells, pretreatment, preconditioning, mesenchymal stromal/stem cells, secretion, regeneration, cytokines

## Abstract

Stem cell-based therapies require cells with a maximum regenerative capacity in order to support regeneration after tissue injury and organ failure. Optimization of this regenerative potential of mesenchymal stromal/stem cells (MSC) or their conditioned medium by in vitro preconditioning regimens are considered to be a promising strategy to improve the release of regenerative factors. In the present study, MSC were isolated from inguinal adipose tissue (mASC) from C57BL/6 mice, cultured, and characterized. Then, mASC were either preconditioned by incubation in a hypoxic environment (0.5% O_2_), or in normoxia in the presence of murine epidermal growth factor (EGF) or tumor necrosis factor α (TNFα) for 48 h. Protein expression was measured by a commercially available array. Selected factors were verified by PCR analysis. The expression of 83 out of 308 proteins (26.9%) assayed was found to be increased after preconditioning with TNFα, whereas the expression of 61 (19.8%) and 70 (22.7%) proteins was increased after incubation with EGF or in hypoxia, respectively. Furthermore, we showed the proliferation-promoting effects of the preconditioned culture supernatants on injured epithelial cells in vitro. Our findings indicate that each preconditioning regimen tested induced an individual expression profile with a wide variety of factors, including several growth factors and cytokines, and therefore may enhance the regenerative potential of mASC for cell-based therapies.

## 1. Introduction

Mesenchymal stromal/stem cells (MSC) are multipotent stromal cells, which have been identified throughout the whole body as immature cells. MSC represent a rare population in the perivascular niche within fully specialized tissues [1]. Classically, MSC are isolated from the bone marrow, but also from nearly all adult tissues, for example adipose tissue and solid organs [2,3]. Cultured MSC release a number of factors in the culture supernatant that improve regeneration in injured organs or tissue [4]. The organ-protective effects of MSC or their conditioned medium (CM) have been investigated in the last decade, demonstrating that either infused stem cells or their CM facilitated tissue and organ recovery predominantly by released regeneration-promoting factors. Multiple pathways might mediate the release of soluble mediators, extracellular vesicles, organelle transfer, or cell-to-cell contacts [5,6,7,8]. Nevertheless, the mechanisms by which MSC enhance regeneration and ease inflammation and injury are not completely understood. Factors that limit the regenerative capacity and the therapeutic efficacy of transplanted MSC are their poor migration into the target tissue. In addition, the transplantation of cells is impeded by high rates of cell death of transplanted cells. Novel strategies to optimize survival and potency, and therefore the regenerative capacity of MSC or their CM, should be the focus of further studies aiming to enhance the regeneration process. To achieve this maximum regenerative capacity of transplanted MSC, the development of new strategies to improve the release of regeneration-promoting factors, and therefore the regenerative efficiency of MSC, is urgently needed.

The comprehensive profiling of factors released by MSC revealed that their secretome consists of various cytokines, chemokines, growth factors, extracellular matrix proteins, RNAs, and molecules involved in vascularization and hematopoiesis. Recent data indicate that the regenerative potential of MSC could be boosted by in vitro pretreatment regimens (“preconditioning”) using environmental or pharmacological stimuli, enhancing their therapeutic efficacy. The factors and vesicles released by pretreated MSC are manifold and exert immunomodulatory, anti-apoptotic, pro-angiogenic, and trophic effects [7]. Currently used in vitro preconditioning regimens for MSC include their culture in a hypoxic or anoxic atmosphere, incubation with trophic factors (growth factors, cytokines, or hormones), application of lipopolysaccharides or pharmacological agents, as well as overexpression of specific factors by genetic modification of the cells [8,9,10,11]. Nevertheless, genetic modifications such as overexpression of genes involved in migration, apoptosis, or survival can be complex to translate into clinical-grade protocols. Therefore, alternative preconditioning regimens without active genetic manipulation should be favored in the development of in vivo applications in humans.

Accordingly, the present study investigated the potential of three different preconditioning regimens—using either a microenvironmental stimulus, a cytokine, or a growth factor—to enhance the release of factors, and therefore the regenerative potential, of murine adipose-derived MSC (mASC). We firstly evaluated the release of 308 proteins into the cell culture medium by applying a commercially available protein array. Then, we validated the effect of these different preconditioning regimens on the mRNA expression of selected factors, three growth factors, two cytokines, and one matrix metalloprotease, by quantitative real-time polymerase chain reaction (qPCR) analysis.

## 2. Results

### 2.1. Characterization of mASC

**Figure 1:**
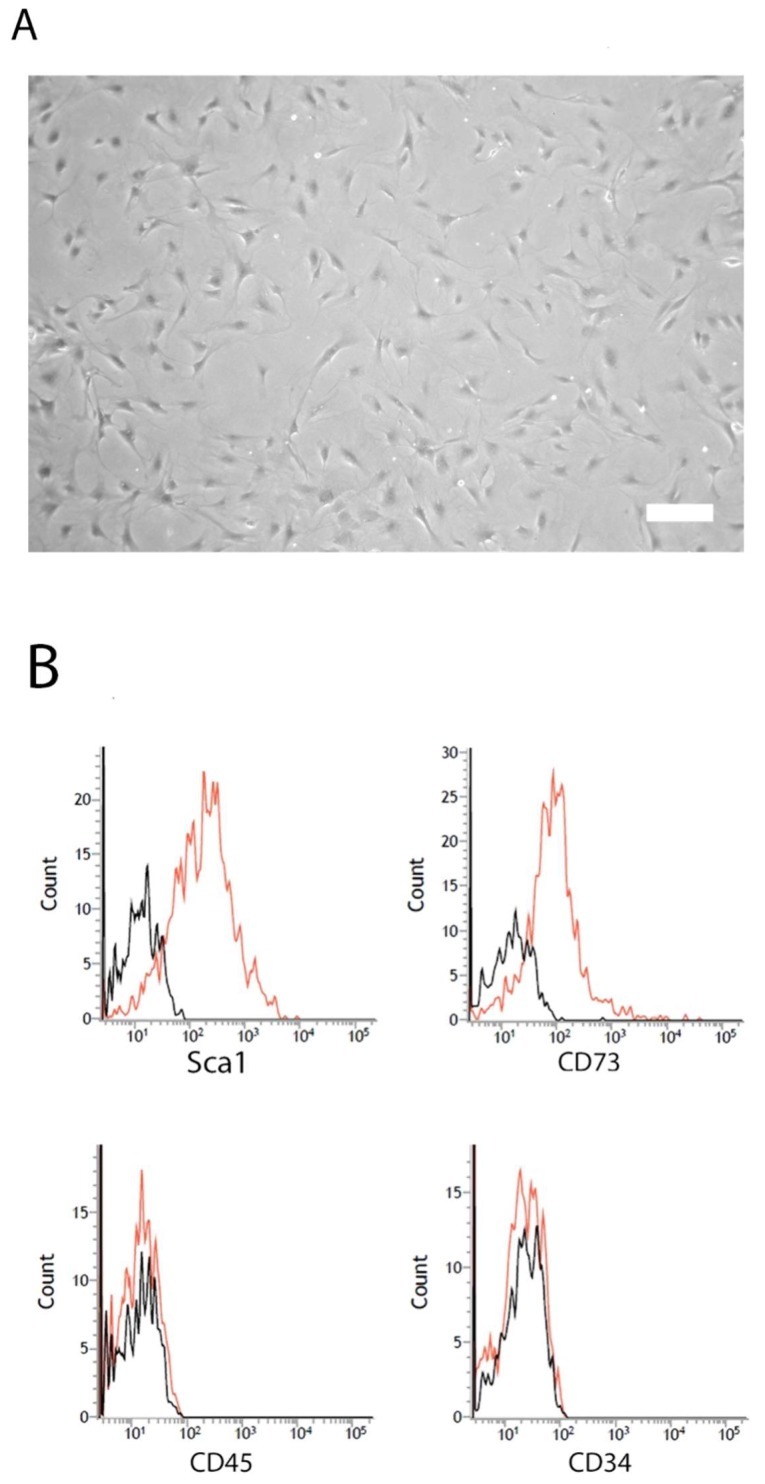
Cell characterization. (**A**) Characteristic phase contrast microscopy of murine adipose-derived stromal/stem cells (mASC) cultured in standard cell culture (bar: 100 µm); (**B**) representative flow cytometric histograms (red) of the expression of characteristic markers. Black histograms show isotype controls.

Cells were characterized by flow cytometric analysis utilizing characteristic markers for murine ASCs. Cultured mASC displayed a spindle-shaped fibroblastoid morphology in culture (Figure 1A). Cultured mASC expressed Sca-1 and CD73, but did not express CD45 or CD34 (Figure 1B). Furthermore, cultured mASC were positive for CD90 and CD105 [12] and underwent differentiation upon stimulation with adipogenic, chondrogenic, and osteogenic media (tri-lineage differentiation) (Supplementary Figure S1).

### 2.2. Measurement of Cell Viability after Preconditioning

**Figure 2:**
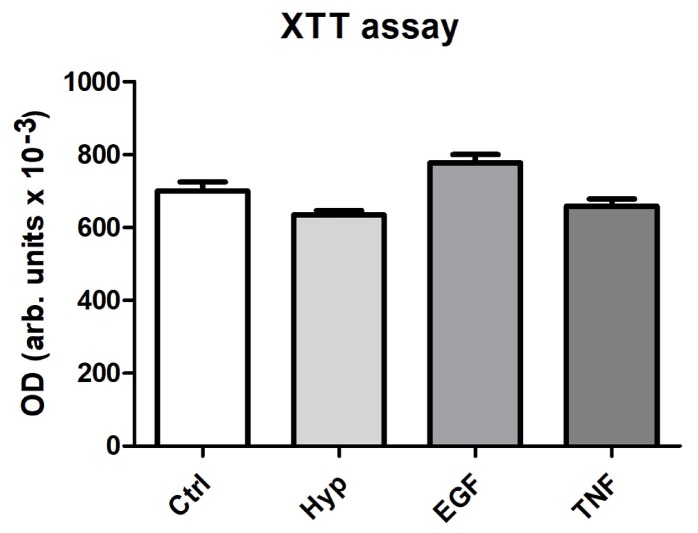
Cell viability after preconditioning for 48 h. Murine ASC were cultured in 96-well plates and preconditioned for 48 h. The XTT assay was performed and optical density (OD) was measured in a microplate reader at 490 nm vs. 650 nm (arbitrary units, mean ± SD, *n* = 5). No significant effects of the different pretreatments on the cell viability could be detected.

We used a commercial assay using 2,3-bis-(2-methoxy-4-nitro-5-sulfophenyl)-2*H*-tetrazolium-5-carboxanilide (XTT) to investigate cell viability after the preconditioning regimens. The assay is a colorimetric assay used to determine cell viability as a function of cell number based on the metabolic activity of the cultured cells. Using this assay we could show that the pretreatment regimens induced no differences in cell viability compared to the control (Figure 2).

### 2.3. Effects of Preconditioning Regimens

**Figure 3:**
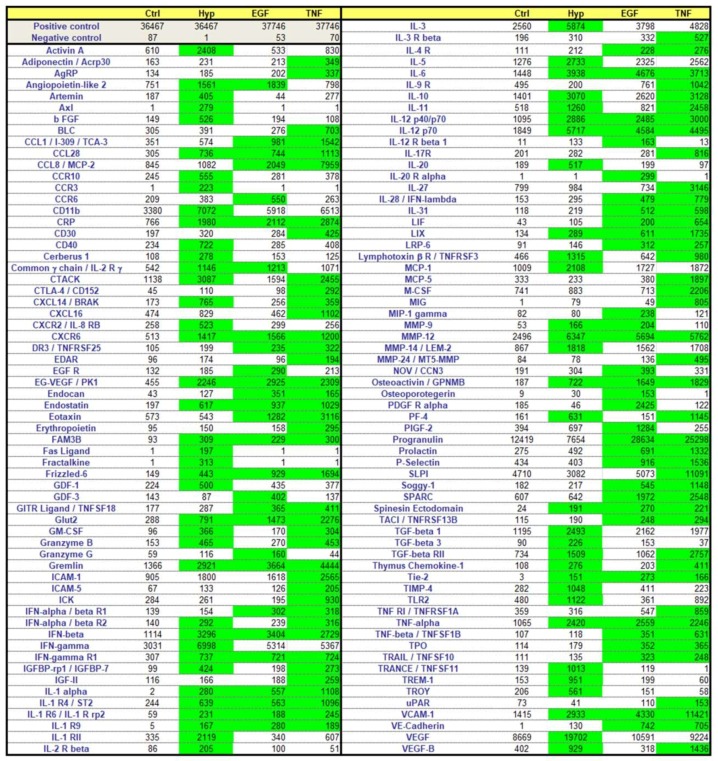
Color map of increased expressed proteins after the preconditioning regimens. Cells were either cultured under standard conditions (Ctrl) or preconditioned by incubation in a hypoxic environment (Hyp; 0.5% O_2_) or in the presence of murine epidermal growth factor (EGF) (10 ng/mL) or murine tumor necrosis factor α (TNFα) (10 ng/mL) for 48 h. The expression of 308 proteins was measured in the cell supernatant after preconditioning by a commercially available protein array. The heatmap displays proteins enhanced at least >2-fold versus the control and >150 arbitrary units after pretreatment (green).

Based on the assumption that soluble factors secreted from mASC represent a major mechanism enhancing tissue and organ regeneration, we investigated the potency of three different pretreatment regimens on the release of regeneration-promoting factors into the culture medium. For this purpose, mASC were cultured in hypoxia (0.5% O_2_) or in the presence of either murine epidermal growth factor (EGF) (10 ng/mL) or murine tumor necrosis factor α (TNFα) (10 ng/mL) for 48 h. By means of a commercially available protein array, 308 mouse proteins were simultaneously detected in the culture supernatant of the preconditioned cells and subsequently compared to cells cultured under normal cell culture conditions (control). Data from this array were analyzed, whereas only values (arbitrary units), which increased more than 2-fold compared to the controls were considered as induced (Figure 3 and Table S1). Furthermore, we did not use values <150 (arbitrary units, after pretreatment). The expression of 83 of the 308 proteins (26.9%) assayed was found to be more than 2-fold increased after preconditioning with TNFα, while the expression of 61 (19.8%) and 70 (22.7%) proteins was increased after incubation with EGF or in hypoxia, respectively (Figure 3).

The factors induced were multifarious; many of them are growth factors (e.g., vascular endothelial growth factor (VEGF), basic fibroblast growt factor (bFGF), insulin-like growth factor-II (IGF-II)), chemokines (e.g., MCP-1, -5, CCL-2), and cytokines (e.g., interferon γ (IFNγ), several interleukins and their receptors), but also matrix metalloproteases (e.g., MMP-9, -12, -14, -24) and adhesion molecules (e.g., ICAM-1 and -5, VE-cadherin, P-selectin, vascular cell adhesion protein (VCAM)). Moreover, the expression intensities were extremely varied between the proteins checked—some of them were also expressed by non-preconditioned mASC, and some of them were de novo expressed after a pretreatment regimen. Complete results from all 308 proteins are shown in a supplementary online table (Table S1).

**Figure 4:**
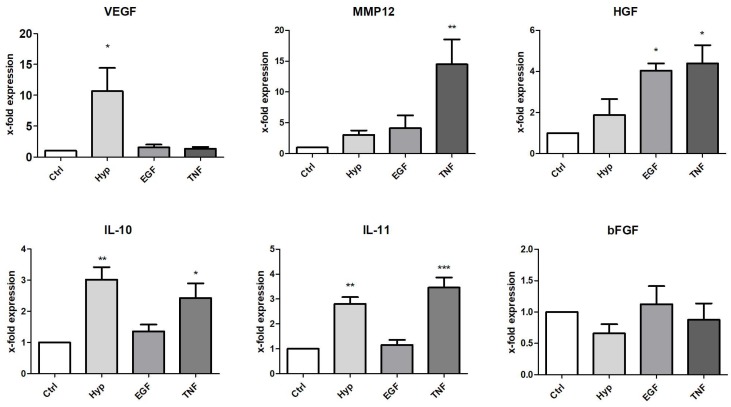
Effect of preconditioning on mRNA expression of selected factors. Expression was measured in total RNA from mASC after preconditioning by culture under hypoxia (Hyp, 0.5% O_2_), or in medium containing EGF (10 ng/mL) or TNFα (10 ng/mL). The expression levels in each experiment were normalized to a housekeeping gene (β-actin) and are expressed relative to the control using the ∆∆CT method. * *p* < 0.05, ** *p* < 0.01, *** *p* < 0.001 versus control, *n* = 4–6.

In order to verify selected factors of the protein array, we quantified the mRNA expression levels of preconditioned cells by qPCR analysis. For this, we used six readouts, and five of them were also increased in the protein array. We further tested one growth factor (hepatocyte growth factor (HGF)), a factor not increased in the protein array, but described to be involved in regeneration. By comparing the results of the array with the qPCR analysis, we identified comparable induction patterns of VEGF, IL-10, and IL-11 mRNA (Figure 4) and protein (Figure 3) after 48 h of preconditioning. RNA expression levels of two factors (bFGF, HGF) differed to some extent from the results from the protein array. PCR results from bFGF expression showed no mRNA induction (Figure 4), whereas the protein expression was increased by Hyp (Figure 3). On the other hand, HGF mRNA, but not protein expression, was significantly induced after incubation in the presence of EGF or TNFα (Figure 4). Regarding MMP12, we found slightly differing results between the two assays. MMP12 protein was significantly induced by all three pretreatments, but mRNA only showed a significant increase after incubation with TNF. MMP12 mRNA was also induced in Hyp and in the presence of EGF (3.06-fold and 4.13-fold, respectively), whereas no statistical significance could be calculated. Nevertheless, the tendencies of MMP12 mRNA and protein measurements were related, and MMP12 mRNA was induced after all three pretreatments.

### 2.4. Effects of Preconditioned Culture Supernatants on Epithelial Cell Proliferation

**Figure 5:**
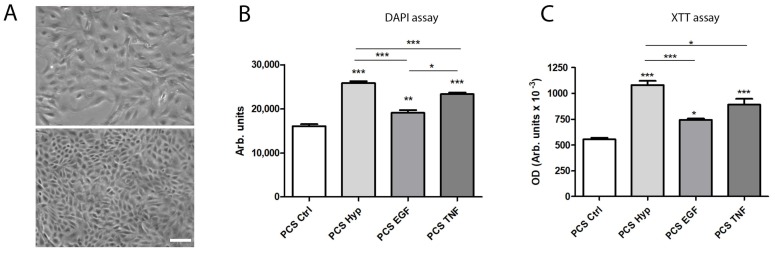
Effect of processed culture supernatant (PCS) after preconditioning (by Hyp, EGF, or TNF) or control culture (Ctrl) on the proliferation and viability of epithelial cells. (**A**) Characteristic phase contrast microscopy of subconfluent (above) and confluent (below) murine renal tubular epithelial cells in culture (bar: 100 µm); (**B**) DAPI assay: cell proliferation was determined by a fluorometric assay using 4,6-diamino-2-phenylindole (DAPI), measuring the DNA content as an indirect determination of proliferation, after incubation with PCS for 72 h. Fluorescence was measured in a fluorescence reader (355 nm ex/460 nm em), and expressed as arbitrary units (mean ± SD, *n* = 6); (**C**) XTT assay: cell viability of mTEC was measured after incubation with PCS for 72 h. The XTT assay was performed and optical density (OD) was measured in a microplate reader at 490 nm vs. 650 nm (arbitrary units, mean ± SD, *n* = 6). * *p* < 0.05, ** *p* < 0.01, *** *p* < 0.001 versus control and among each preconditioning regimen.

Subsequent to the analysis of the increased expression of different factors after mASC preconditioning, we further evaluated the proliferation-promoting effects of the culture supernatants on the regeneration of murine renal tubular epithelial cells (mTEC) in vitro. Therefore, we processed the preconditioned culture supernatants (PCS) from preconditioned mASC (and from mASC in standard culture), and then incubated these supernatants with subconfluent, injured mTEC for 72 h. Using the DNA content as an indirect measurement of cell proliferation, we showed that factors released after all three pretreatment regimens significantly induced mTEC proliferation (Figure 5B). In addition, the XTT assay showed an increased cell viability/proliferation after incubation with all three PCS (Figure 5C). This assay is not only used as a viability assay, but also standardly used as a proliferation assay. The comparison of the regimens also revealed significant differences. PCS from hypoxia-preconditioned mASC significantly induced a higher induction of mTEC proliferation compared to the two other pretreatments (Figure 5B,C).

## 3. Discussion

Adult adipose-derived stromal/stem cells are multipotent cells with a strong capability to release various factors with regeneration-promoting, immunomodulatory, anti-fibrotic, pro-angiogenic, and anti-apoptotic potential [7]. They are therefore promising cells for regenerative medicine and cell therapy. The intercellular crosstalk between transplanted and resident cells is mainly realized by soluble factors as well as extracellular vesicles/exosomes released from transplanted cells. Both mechanisms are described as key factors in regeneration and repair. Recent data indicate that this potential could be boosted by pretreatment (or preconditioning) with environmental or pharmacological stimuli, also enhancing their therapeutic efficacy. The secretome (or the paracrine profile) of pretreated MSC seems to vary according to the respective preconditioning regimen. Different pretreatment regimens either activate or suppress varying molecular signals and signal transduction cascades. The cellular responses are very complex, since the preconditioning regimen affects a great number of factors and not only a single, specific factor. The trophic factors released by pretreated cells are manifold, but the protein expression profiles after pretreatment regimens are not comprehensively characterized. Therefore, the rationale of our recent study was to characterize the expression profile of mASC after different short-term in vitro preconditioning regimens and to prove the effects of the factors induced in an in vitro model of cell regeneration.

Several potential strategies have been explored to enhance the paracrine potency of MSC and thus their therapeutic efficacy before administration into in vivo models or clinical studies. Hypoxic preconditioning has been described to enhance cell proliferation and angiogenic potential, as well as the survival of MSC [13,14,15,16]. It also leads to metabolic changes resulting in higher in vivo cell survival after transplantation [14], and induces the expression of factors that are involved in migration and homing [17]. For example, Lee and co-workers showed that MMP12 is involved in the migration of MSC [18]. In our study, MMP12 protein expression was significantly increased in all three preconditioning regimens.

Hypoxic preconditioning protects MSC through the activation of anti-apoptotic signaling mechanisms and enhances their angiogenic potential by the induction of proangiogenic factors in vitro [19]. The downstream signaling pathway during hypoxia causes the induction and translocation of HIF1α to the cell nucleus, which in turn mediates the expression of regeneration-promoting genes (e.g., *VEGF*) [16]. Accordingly, VEGF mRNA and protein were also significantly increased by hypoxia in the present study. In this context, it has also been shown that the hypoxic preconditioning of MSC promotes the release of angiogenic cytokines and improves the survival of the transplanted cells in an in vivo model of hindlimb ischemia [20]. We recently investigated the regeneration-promoting effects of medium from Hyp-preconditioned mASC in an in vivo model of acute kidney injury [13]. In this study, we verified that medium from Hyp-pretreated mASC significantly ameliorated levels of serum creatinine, neutrophil gelatinase-associated lipocalin, and inflammatory cytokines IL-1β and IL-6 in the serum of mice during acute kidney injury. This work further demonstrated that hypoxic pretreatment enhanced the therapeutic effects and survival of mice with cisplatin-induced acute kidney injury [13].

Another approach to increase the therapeutic potential and regenerative capacity of MSC constitutes the preconditioning with growth factors, cytokines, or other small molecules (reviewed in Reference [6]). For example, EGF has been shown to promote in vitro expansion of MSC without altering their multipotency [21,22], but enhances MSC motility and migration [21,23]. Furthermore, a functional EGF receptor was identified on MSC with evidence of active EGF signal transduction [23]. Interestingly, we detected an increased protein expression of the EGF-receptor after EGF pretreatment. The pretreatment of MSC with EGF has previously been described to enhanced the release of factors like VEGF, HGF, heparin-binding EGF like growth factor (HB-EGF), and interleukin-6 and -11, but not FGF-2 [22,24]. Nevertheless, we were not able to show a significant induction of VEGF mRNA (1.55-fold) and also detected only a slight increase in VEGF protein expression. In contrast to VEGF, we could show a significant induction of HGF mRNA expression after EGF pretreatment, however not of HGF protein. VEGF and HGF are described to play a pivotal role in MSC-mediated accelerated wound healing by inducing angiogenesis and improving oxygen supplies to ischemic tissues [22].

Pretreatment by TNFα is also described to increase MSC’s release of cytokines, chemokines, and proteases compared to untreated MSC. Lee and co-workers identified the enhanced release of 118 proteins into the culture supernatant upon TNFα incubation [25]. Many of them, for example IL-6, IL-8, and MCP-1, are known to be critically involved in inflammatory processes. Likewise, we detected numerous pro- and anti-inflammatory cytokines and chemokines such as IL-6, IL-10, IL-11, IL-12, MIG/CXCL9, CXCL16, MCP-2/CCL8, and CCL1 to be induced after TNF pretreatment. Inflammation is known to be a key response to injury, with cytokines and chemokines being associated with regeneration processes.

In addition, we tested the effects of the released factors on the proliferation and viability of injured epithelial cells. Epithelial cells cultured in a subconfluent state resemble wounded or injured epithelia rather than healthy and well-differentiated epithelia [26]. Depending on the isolation and culture procedure, epithelial cells in vitro show partial loss of their polarity and intercellular junctions as well as acquisition of mesenchymal characteristics [26]. In vivo, this may also occur during organ or tissue injury when parts of the epithelial layer are lost, either by apoptosis or necrosis. Therefore, we applied this in vitro model of cell regeneration and showed the significantly increased proliferation of mTEC in processed pretreated mASC supernatant compared to supernatant from non-pretreated mASC. The assays also clearly showed that preconditioning in a hypoxic microenvironment significantly induced the strongest effect on mTEC proliferation (Figure 5B,C).

In summary, our recent study characterized the secretome of induced mASC pretreated by three different preconditioning regimens. The work clearly showed that ASCs can be stimulated effectively to secrete factors by all three regimens used. Yet we were able to achieve an individual pattern of secreted growth factors, matrix metalloproteases, adhesion molecules, chemokines and cytokines, and receptors for each regimen (Figure 3, Supplementary Table S1). Furthermore, the proliferation-promoting effects of the factors released after the pretreatments were clearly shown by the in vitro assays using wounded epithelial cells.

In conclusion, an in vitro preconditioning regimen represents a promising strategy for regenerative therapies, not only to enhance the paracrine profile and regenerative capacity of MSC, but maybe also to decrease the number of cells for transplantation and, therefore, to reduce the risk of side effects. Nevertheless, further in vivo studies are needed to demonstrate the effect of each pretreatment in vivo and to evaluate the best preconditioning regimen for each specific application. A thorough examination of MSC’s secretome and the development of new strategies to improve the release of regeneration-promoting factors seem to be essential for an optimal therapy design in human regenerative medicine. In this case, several questions remain concerning the effects of preconditioning on MSC’s secretome and their regeneration-promoting functions in human regenerative medicine. However, harnessing the secretome of MSC for human regenerative medicine will likely be realized in the near future.

## 4. Materials and Methods

### 4.1. Animals

Eleven-week-old female C57BL/6 mice (Janvier, Saint-Berthevin Cedex, France) with free access to food and water were caged in a room featuring a 12-h light/darkness rhythm. All animal procedures were approved by the Animal Care and Use Committee of the state of Hessen (RP Darmstadt, permit number F61/19, 16 July 2012). Animal care and procedures were performed in accordance with the “Guide for the care and use of laboratory animals” (NIH, volume 25, no. 28, revised 1996), EU Directive 86/609, and the German Animals Protection Act.

### 4.2. Cell Isolation and Culture

Adipose tissue was harvested from C57BL/6 mice. Mice were killed by cervical dislocation. Adipose tissue from inguinal fat pads was immediately dissected to isolate adipose-derived stromal/stem cells (mASC). In brief, tissue was minced with two scalpels (crossed blades) and then incubated in a 0.5% collagenase/phosphate buffered saline (PBS) solution (Collagenase Type: CLS, Biochrom, Berlin, Germany; PBS, Sigma, Taufkirchen, Germany) for 1 h at 37 °C under constant shaking. The digested tissue solution was then separated through a 100-µm strainer and the resulting filtrate was centrifuged at 300× *g* for 5 min. The resulting pellet was washed twice with medium and centrifuged again at 300× *g* for 5 min. Finally, cells were plated for initial cell culture and cultured at 37 °C in an atmosphere of 5% CO_2_ in humid air. Dulbecco’s modified Eagle’s medium (DMEM, Sigma, Taufkirchen, Germany) with a physiologic glucose concentration (100 mg/dL) was supplemented with 10% fetal calf serum (Biochrom, Berlin, Germany) and used as a standard culture medium. The medium was replaced every three days. Subconfluent cells (90%) were passaged by trypsinization. Cells between passages 2 and 5 were used throughout the experiments. Cell morphology was examined by phase contrast microscopy. Flow cytometric analysis was carried out to show the characteristic marker expression of cultured mASC. Cells were detached from the cell culture plastic and stained with directly labeled antibodies (Sca-1-APC (eBioscience, San Diego, CA, USA), CD73-PE-Cy7 (BD Bioscience, Heidelberg, Germany), CD34-PE (Immunotools, Friesoythe, Germany), and CD45-FITC (Immunotools, Friesoythe, Germany)). The labeled cells were analyzed using a flow cytometer (BD Biosciences, Heidelberg, Germany). All experiments included negative controls with corresponding isotype controls. A flow cytometer was set using isotype controls. Cells were gated by forward and sideward scatter to eliminate debris. The tri-lineage differentiation potential of cultured mASC was induced by incubation in differentiation media for 14 days followed by the verification of differentiation by standard staining methods (Oil-O-Red, Alcian Blue, and von Kossa staining, respectively), as further described [27].

Murine renal tubular epithelial cells (mTEC) were isolated by protocols described earlier, with modifications [28,29]. In brief, murine kidneys harvested from C57BL/6 mice were washed extensively with sterile PBS to remove contaminating debris and red blood cells. Kidneys were then minced into approximately 1-mm^2^ pieces, and digested with 0.1% collagenase diluted in PBS for 10 min at 37 °C with gentle agitation. The collagenase was inactivated with an equal volume of culture medium, and centrifuged for 5 min at 300× *g*. The cellular pellet was resuspended in culture medium and sequentially filtered through 70- and 40-µm mesh filters to remove debris. Cells remaining in the 40-µm filter were used, washed, and cultured in culture medium. Medium 199 (M4530, Sigma, Taufkirchen, Germany) with a physiologic glucose concentration (100 mg/dL) was supplemented with 10% fetal calf serum (Biochrom, Berlin, Germany) and used as culture medium for mTEC. The medium was replaced every three days. Confluent cells were passaged by trypsinization. Cells between passages 2 and 4 were used for the experiments.

### 4.3. Preconditioning Regimens

Cells were either cultured under standard conditions (controls) or preconditioned by incubation in a hypoxic environment (Hyp) or in the presence of recombinant murine epidermal growth factor (EGF, No. 12343406, Immunotools, Friesoythe, Germany) or murine tumor necrosis factor-alpha (TNFα, No. 123430146, Immunotools, Friesoythe, Germany). Cells treated with hypoxia were placed in an InvivO_2_ 400 (Baker and Baker Ruskinn, Sanford, FL, USA) at 0.5% oxygen. TNFα or EGF was added to serum-free DMEM in a final concentration of 10 ng/mL. Controls were cultured in serum-free DMEM. All treatments were performed for 48 h in serum-free low-glucose DMEM. Afterwards, the medium was removed and centrifuged at 300× *g* for 10 min or processed as described below.

### 4.4. Cell Viability and Proliferation Assays

To determine cytotoxic effects during the preconditioning regimens, cell viability of mASC was determined by a photometric assay using 2,3-Bis-(2-Methoxy-4-Nitro-5-Sulfophenyl)-2*H*-Tetrazolium-5-Carboxanilide (XTT), as described previously [30]. In brief, subconfluent mASC in 96-well plates were preconditioned for 48 h as described above. Afterwards, XTT reagent was added to wells as described by the manufacturer (AppliChem, Darmstadt, Germany) and incubated at 37 °C. Absorbance was measured in a microplate reader at 490 nm vs. 650 nm.

The effects of the supernatants from the preconditioning regimens on epithelial cell proliferation were determined by a fluorometric assay using 4,6-diamino-2-phenylindole (DAPI), measuring the DNA content as an indirect determination of cell number and proliferation [31]. In brief, cells were permeabilized using 0.02% sodium dodecyl sulphate (SDS), 150 mM NaCl and 15 mM sodium citrate. Finally, DAPI (2 μg/mL) was added to each well. Fluorescence was measured in a fluorescence reader (355 nm ex/460 nm em, FluoStar, BMG Labtech, Offenburg, Germany). Furthermore, viability of mTEC was measured by the XTT assay, as described above.

### 4.5. Protein Array

We used a commercially available protein array (Tebu-Bio, Offenbach, Germany, No. AAM-BLG-1-4) for the simultaneous detection of the relative expression of 308 murine proteins in the cell culture supernatant. The cells were preconditioned as described above. All supernatants were collected after preconditioning for 48 h and centrifuged for 10 min at 1000× *g*. Then, the protein content of the supernatants was determined by a routine method using bicinchoninic acid (BCA). Supernatants were 6.6-fold concentrated using a 3 kDa molecular weight cut-off Amicon Ultra-4 filter (No. UFC800324, Merck Millipore, Darmstadt, Germany). Finally, the protein array was processed according to the manufacturer’s protocol. The readily prepared array was then sent to the manufacturer, which performed the analysis of the array.

### 4.6. PCR

Total RNA was isolated immediately after preconditioning for 48 h. RNA extraction was performed using single-step RNA isolation from cultured cells by a standard protocol. After RNA extraction, cDNAs were synthesized for 30 min at 37 °C using 1 µg RNA, 50 µM random hexamers, 1 mM deoxynucleotide-triphosphate-mix, 50 units of reverse transcriptase (Fermentas, St. Leon-Rot, Germany) in 10× PCR buffer, 1 mM β-mercaptoethanol, and 5 mM MgCl_2_. An Absolute qPCR SYBR Green Rox Mix was used (Thermo Scientific, Hamburg, Germany) for the master mix; primer mix and RNAse-free water were added. Quantitative PCR was carried out in 96-well plates using the following conditions: 15 min at 95 °C for enzyme activation, 15 s at 95 °C for denaturation, 30 s for annealing, and 30 s at 72 °C for elongation. Lastly, a melting curve analysis was conducted. Products were checked by agarose gel electrophoresis in selected experiments. Quantification of the PCR fragment was carried out using the Eppendorf realplex^2^ Mastercycler epgradient S (Eppendorf, Hamburg, Germany). Relative quantification was estimated by the ∆∆CT method [32] with β-actin as a calibrator. The level of target gene expression was calculated using 2^−∆∆*C*t^. In addition, PCR products were separated by agarose electrophoresis and observed under UV illumination. Primer pairs were synthesized by Invitrogen (Karlsruhe, Germany) and are listed in Table 1.

### 4.7. Effect of Preconditioned Medium on the Proliferation of mTEC

To determine regenerative effects of processed supernatants from preconditioned mASC (versus supernatant from mASC in standard culture), we quantified the growth-promoting effects of the supernatants of non-confluent and, therefore, injured epithelial cells [28]. Supernatants from mASC after the preconditioning regimens (and from non-pretreated controls) were collected and centrifuged for 10 min at 1000× *g*. Then, supernatants were 6.6-fold concentrated using a 3 kDa molecular weight cut-off Amicon Ultra-4 filter (No. UFC800324, Merck Millipore, Darmstadt, Germany), and 10-fold diluted in medium 199 (No. M4530, Sigma, Taufkirchen, Germany) to replace glucose and all other essential culture ingredients. In the next step, 10,000 mTEC were seeded in 96-well plates and cultured for two to three days. Then, mTEC were serum-depleted for 2 h and incubated in the presence of PCS for 72 h. Proliferation and viability were estimated as described above.

### 4.8. Statistical Analysis

The data were expressed as means ± standard deviation (SD). Analysis of variance (ANOVA) with Bonferroni’s Multiple Comparison Test was used for statistical analysis. *p* values < 0.05 were considered significant.

## Figures and Tables

**Table 1:** Primer used for qPCR analyses.

Gene	Primer Forward	Primer Reverse	Product Length (bp)	NCBI Reference Sequence
*VEGF*	ATG AAC TTT CTG CTC TCT TG	CTT CTG CTC TCC TTC TGT C	105	NM_001025250
*bFGF*	AAC TAC AAC TCC AAG CAG AA	CGT TCA AAG AAG AAA CAC TC	136	NM_008006
*IL-11*	CTT CAG ACC CTC GAG CAG AT	CGT CAG CTG GGA ATT TGT CT	108	NM_008350.4
*IL-10*	TCC CCT GTG AAA ATA AGA G	CAG TTG ATG AAG ATG TCA AA	112	NM_010548.2
*MMP12*	CTC TGC TGA AAG GAG TCTG	AAT TCT GTC CTT TCC ATA ATC	146	NM_008605
*HGF*	CCT TTG CTT TGA TTC TTTC	TTC TTC TTT TCT TCT GTC CTT	177	NM_001289458
*β-Actin*	CCA CCA TGT ACC CAG GCA TT	AGG GTG TAA AAC GCA GCT CA	253	NM_007393

## Supplementary Materials

Supplementary materials can be found at http://www.mdpi.com/1422-0067/19/6/1719/s1.

## Abbreviations

bFGF	Basic fibroblast growth factor
CM	Conditioned medium
EGF	Epidermal growth factor
HGF	Hepatocyte growth factor
Hyp	Hypoxic environment
IL	Interleukin
mASC	Murine adipose-derived stromal/stem cells
MMP12	Matrix metalloproteinase-12
MSC	Mesenchymal stromal/stem cells
PCR	Quantitative real-time polymerase chain reaction
TNFα	Tumor necrosis factor-alpha
VEGF	Vascular endothelial growth factor
