# Supplementary material for: Effect of Different Preconditioning Regimens on the Expression Profile of Murine Adipose-Derived Stromal/Stem Cells
Source: Int J Mol Sci. 2018 Jun 10;19(6):1719. doi: 10.3390/ijms19061719 (PMC6032282; doi:10.3390/ijms19061719)
Supplement: Supplementary file 1 [file ijms-19-01719-s001.pdf]

Supplementary Materials: Effect of Different Preconditioning Regimens on the Expression Profile of Murine Adipose-Derived Stromal/Stem Cells

Patrick C. Baer, Jürgen M. Overath, Anja Urbschat, Ralf Schubert, Benjamin Koch, Asanke A. Bohn and Helmut Geiger

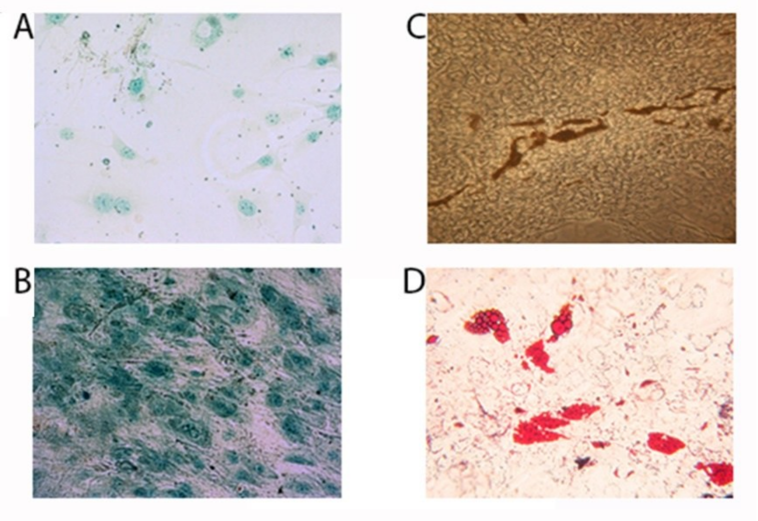

Figure S1. Tri-lineage differentiation potential of cultured mASC. Differentiation of cultured mASC was induced by incubation in differentiation medium for 14 days, followed by standard staining methods (50x magnification). (**A**,**B**) Alcian Blue staining of undifferentiated controls (**A**) and differentiated mASC (**B**) after 14 days. Chondrogenic differentiation was induced by medium containing 0.22 mM ascorbic acid, 6.25 μg/mL insulin, and 10 ng/mL TGF-β and proven by Alcian Blue staining; (**C**) Von Kossa staining. Osteogenic differentiation was induced using medium containing 0.1 μM dexamethasone, 50 μM ascorbic acid, and 10 mM β-glycerophosphate and proven by von Kossa staining. Undifferentiated controls were negative ; (**D**) Oil-O-Red staining. Adipogenic differentiation was induced by medium containing 0.5 mM isobutyl-methylxanthine, 1 μM dexamethasone, 200 μM indomethacin, 1.74 μM insulin, 3500 mg/L glucose and proven by Oil-O-Red staining. Undifferentiated controls were negative.

Table S1. Complete data of the 308 proteins measured by the commercially available protein array.

	Ctrl	Hyp	EGF	TNF
**Positive Control**	36467	36467	37746	37746
**Neg**	87	1	53	70
6Ckine	234	397	356	380
Activin A	610	2408	533	830
Activin C	263	426	423	306
Activin RIB / ALK-4	1	39	21	1
Adiponectin / Acrp30	163	231	213	349
AgRP	134	185	202	337
ALCAM	152	191	209	169
Angiopoietin-like 2	751	1561	1839	798
Angiopoietin-like 3	197	229	198	349
AR (Amphiregulin)	345	444	332	397
Artemin	187	405	44	277
Axl	1	279	1	1
b FGF	149	526	194	108
B7-1/CD80	1	147	10	3
BAFF R / TNFRSF13C	100	85	1	1
BCMA / TNFRSF17	118	174	121	47
beta-Catenin	1	1	1	1
BLC	305	391	276	703
BTC (Betacellulin)	1	1	1	1
Cardiotrophin-1	52	33	102	1
CCL1 / I-309 / TCA-3	351	574	981	1542
CCL28	305	736	744	1113
CCL4 / MIP-1 beta	101	153	67	1
CCL7 / MCP-3 / MARC	7657	8323	7518	8196
CCL8 / MCP-2	845	1082	2049	7959
CCR10	245	555	281	378
CCR3	1	223	1	1
CCR4	1	86	1	1
CCR6	209	383	550	263
CCR7	112	167	98	42
CCR9	204	213	203	271
CD11b	3380	7072	5918	6513
CD14	157	215	156	301
CRP	766	1980	2112	2874
CD27 / TNFRSF7	155	214	261	266
CD27 Ligand / TNFSF7	292	419	417	353
CD30	197	320	284	425
CD30 L	243	394	294	426
CD40	234	722	285	408

CD40 Ligand / TNFSF5	309	526	327	437
Cerberus 1	108	278	153	125
Chordin-Like 2	1	1	1	1
Coagulation Factor III / Tissue Factor	201	214	369	358
Common gamma Chain / IL-2 R gamma	542	1146	1213	1071
CRG-2	101	124	109	104
Cripto	232	252	258	394
Crossveinless-2	667	565	563	1048
Cryptic	43	1	11	29
Csk	12	1	58	80
CTACK	1138	3087	1594	2455
CTLA-4 / CD152	45	110	98	292
CXCL14 / BRAK	173	765	256	359
CXCL16	474	829	462	1102
CXCR2 / IL-8 RB	258	523	299	256
CXCR3	189	208	140	153
CXCR4	1	1	11	1
CXCR6	513	1417	1566	1200
DAN	1	1	1	1
Decorin	169	121	157	1
DKK-1	12	1	1	1
Dkk-3	22	89	106	1
Dkk-4	191	117	180	229
DPPIV / CD26	1	1	42	1
DR3 / TNFRSF25	105	199	235	322
Dtk	567	595	608	547
EDAR	96	174	96	194
EGF R	132	185	290	213
EG-VEGF / PK1	455	2246	2925	2309
Endocan	43	127	351	165
Endoglin / CD105	248	360	281	317
Endostatin	197	617	937	1029
Eotaxin	573	543	1282	3116
Eotaxin-2	4539	3777	5167	3401
Epigen	239	264	349	403
Epiregulin	1	1	1	1
Erythropoietin (EPO)	95	150	158	295
E-Selectin	214	337	299	356
FADD	117	118	56	100
FAM3B	93	309	229	300
Fas / TNFRSF6	212508	207556	172285	128401
Fas Ligand	1	197	1	1
FCrRIIB / CD32b	252	409	244	162

FGF R3	223	272	299	365
FGF R4	32	81	106	9
FGF R5 beta	1024	1950	1795	1686
FGF-21	1	1	1	1
Fit-3 Ligand	1	1	1	1
FLRG (Follistatin)	103	2	39	7
Follistatin-like 1	144	215	273	179
Fractalkine	1	313	1	1
Frizzled-1	1	1	1	1
Frizzled-6	149	443	929	1694
Frizzled-7	1	133	1	74
Galectin-3	731	405	707	978
G-CSF	299	534	297	279
GDF-1	224	500	435	377
GDF-3	143	87	402	137
GDF-5	1	91	112	97
GDF-8	712	1302	1014	768
GDF-9	1	1	1	1
GFR alpha-2 / GDNF R alpha-2	200	261	241	246
GFR alpha-3 / GDNF R alpha-3	236	289	291	301
GFR alpha-4 / GDNF R alpha-4	105	86	203	168
GITR	146	161	160	211
GITR Ligand / TNFSF18	177	287	365	411
Glut2	288	791	1473	2276
GM-CSF	96	366	170	304
Granzyme B	153	465	270	453
Granzyme D	120	155	158	38
Granzyme G	59	116	160	44
Gremlin	1366	2921	3664	4444
Growth Hormone R	1	1	38	1
HGF R	190	290	221	217
HGF	2566	3667	3360	2450
HVEM / TNFRSF14	164	167	194	171
ICAM-1	905	1800	1618	2565
ICAM-2 / CD102	463	432	453	574
ICAM-5	67	133	126	205
ICK	284	261	195	930
IFN-alpha / beta R1	139	154	302	318
IFN-alpha / beta R2	140	292	239	316
IFN-beta	1114	3296	3404	2729
IFN-gamma	3031	6998	5314	5367
IFN-gamma R1	307	737	721	724
IGFBP-1	389	465	484	640

IGFBP-2	941	1032	1098	1444
IGFBP-3	205	258	298	258
IGFBP-5	332	579	380	232
IGFBP-6	493	408	371	447
IGFBP-rp1 / IGFBP-7	99	424	198	273
IGF-I	536	507	550	485
IGF-II	116	166	188	259
IL-1 alpha	2	280	557	1108
IL-1 beta	1	1	1	1
IL-1 R4 / ST2	244	639	563	1096
IL-1 R6 / IL-1 R rp2	59	231	188	245
IL-1 R9	5	167	280	189
IL-1 RI	258	414	297	1
IL-1 RII	335	2119	340	607
IL-2	726	726	802	935
IL-2 R alpha	108	154	109	29
IL-2 R beta	86	205	100	51
IL-3	2560	5874	3798	4828
IL-3 R alpha	1	1	10	1
IL-3 R beta	196	310	332	527
IL-4	1	1	1	1
IL-4 R	111	212	228	276
IL-5	1276	2733	2325	2562
IL-5 R alpha	226	449	367	491
IL-6	1448	3938	4676	3713
IL-6 R	1	50	1	1
IL-7	227	265	252	274
IL-7 R alpha	70	89	16	117
IL-9	152	153	41	233
IL-9 R	495	200	761	1042
IL-10	1401	3070	2620	3128
IL-10 R alpha	1	1	1	1
IL-11	518	1260	821	2458
IL-12 p40/p70	1095	2886	2485	3000
IL-12 p70	1849	5717	4584	4495
IL-12 R beta 1	11	133	163	13
IL-13	1	1	1	1
IL-13 R alpha 2	1	1	45	1
IL-15	157	88	112	96
IL-15 R alpha	560	719	494	910
IL-16	714	1280	513	622
IL-17	1800	3567	2673	3326
IL-17BR	186	101	169	154

IL-17C	198	54	102	132
IL-17D	46	1	1	1
IL-17E	189	71	144	127
IL-17F	52	56	35	122
IL-17R	201	282	281	816
IL-17RC	527	708	708	675
IL-17RD	138	158	161	97
IL-18 R alpha/IL-1 R5	15	46	44	1
IL-20	189	517	199	97
IL-20 R alpha	1	1	299	1
IL-21	43	1	1	1
IL-21 R	4	6	12	1
IL-22	171	120	115	263
IL-22BP	93	125	43	34
IL-23	1	1	1	1
IL-23 R	98	50	11	34
IL-24	150	83	63	87
IL-27	799	984	734	3146
IL-28 / IFN-lambda	153	295	479	779
IL-31	118	219	512	598
IL-31 RA	5	83	50	33
Insulin	43	77	105	1
Integrin beta 2 / CD18	49	8	98	1
I-TAC	43	1	1	1
KC	1	2	1	1
Kremen-1	1	1	1	1
Kremen-2	211	119	112	209
Lefty-1	106	40	44	32
Leptin R	106	44	107	126
LEPTIN(OB)	1	1	12	1
LIF	43	105	200	654
LIGHT / TNFSF14	41	4	1	93
LIX	134	289	611	1735
LRP-6	91	146	312	257
L-Selectin	39	34	14	1
Lungkine	197	194	302	224
Lymphotactin	194	81	122	104
Lymphotoxin beta R / TNFRSF3	466	1315	642	980
MAdCAM-1	1	1	1	1
MCP-1	1009	2108	1727	1872
MCP-5	333	233	380	1897
M-CSF	741	883	713	2206
MDC	149	123	103	154

MFG-E8	239	185	202	274
MFRP	103	71	151	205
MIG	1	79	49	805
MIP-1 alpha	1	34	1	1
MIP-1 gamma	82	80	238	121
MIP-2	209	269	276	280
MIP-3 alpha	26	1	38	1
MIP-3 beta	153	43	158	40
MMP-2	10550	10805	18735	12572
MMP-3	32853	35703	56922	52329
MMP-9	53	166	204	110
MMP-12	2496	6347	5694	5762
MMP-14 / LEM-2	867	1818	1562	1708
MMP-24 / MT5-MMP	84	78	136	495
Neuregulin-3 / NRG3	25	1	1	1
Neurturin	49	70	137	119
NGF R / TNFRSF16	106	122	125	206
NOV / CCN3	191	304	393	331
Osteoactivin / GPNMB	187	722	1649	1829
Osteopontin	3214	3618	4855	5208
Osteoporotegerin	9	30	153	1
OX40 Ligand / TNFSF4	159	86	199	191
PDGF C	59	17	79	1
PDGF R alpha	185	46	2425	122
PDGF R beta	201	120	183	166
Pentraxin3 / TSG-14	359	272	447	416
PF-4	161	631	151	1145
PlGF-2	394	697	1284	255
Progranulin	12419	7654	28634	25298
Prolactin	275	492	691	1332
P-Selectin	434	403	916	1536
RAGE	1	1	59	1
RANTES	1	77	48	134
RELM beta	119	97	102	87
Resistin	161	192	212	42
S100A10	103	22	53	1
SCF	204	102	79	1
SCF R / c-kit	109	27	35	1
SDF-1	1	1	1	1
Serum Amyloid A1	321	85	256	102
Shh-N	248	144	207	269
SIGIRR	144	73	108	46
SLPI	4710	3082	5073	11091

Soggy-1	182	217	545	1148
SPARC	607	642	1972	2548
Spinesin Ectodomain	24	191	270	221
TACI / TNFRSF13B	115	190	248	294
TARC	38	116	107	57
TCA-3	356	296	542	422
TCCR / WSX-1	613	948	1083	646
TECK	402	127	248	244
TFPI	1621	1850	2454	2113
TGF-beta 1	1195	2493	2162	1977
TGF-beta 2	167	9	1	1
TGF-beta 3	90	226	153	37
TGF-beta RI / ALK-5	1	39	1	1
TGF-beta RII	734	1509	1062	2757
Thrombospondin	1414	868	917	1548
Thymus Chemokine-1	108	276	203	411
Tie-2	3	151	273	166
TIMP-1	3953	3908	4633	4715
TIMP-2	1764	2083	2055	2566
TIMP-4	282	1048	411	223
TL1A / TNFSF15	146	95	204	122
TLR1	62	80	60	1
TLR2	480	1122	361	892
TLR3	108	36	47	1
TLR4	197	91	114	4
TMEFF1 / Tomoregulin-1	104	94	3	86
TNF RI / TNFRSF1A	359	316	547	859
TNF RII	481	342	453	590
TNF-alpha	1065	2420	2559	2246
TNF-beta / TNFSF1B	107	118	351	631
TPO	114	179	352	365
TRAIL / TNFSF10	111	135	323	248
TRAIL R2 / TNFRSF10B	1	52	39	1
TRANCE / TNFSF11	139	1013	119	1
TREM-1	153	951	199	60
TROY	206	561	151	58
TSLP	224	208	108	164
TSLP R	205	164	230	235
TWEAK / TNFSF12	309	152	303	219
TWEAK R / TNFRSF12	174	124	157	210
Ubiqultin	1153	606	1528	386
uPAR	73	41	110	153
Urokinase	1128	927	1794	1311

VCAM-1	1415	2933	4330	11421
VE-Cadherin	1	130	742	705
VEGF	8669	19702	10591	9224
VEGF R1	196	292	289	184
VEGF R2	209	233	211	129
VEGF R3	184	119	149	178
VEGF-B	402	929	318	1436
VEGF-C	212	151	109	213
VEGF-D	55	5	36	1
WIF-1	1	1	1	1
WISP-1 / CCN-4	287	377	347	510
